# Identification of Chinese Herbal Compounds with Potential as JAK3 Inhibitors

**DOI:** 10.1155/2019/4982062

**Published:** 2019-04-09

**Authors:** Dan Su, Yu-Qiao Gao, Yong-Jie Deng, Han-Hui Zhang, You-Ru Wu, Ying Hu, Quan-Xi Mei

**Affiliations:** ^1^Material and Food College, Zhongshan Institute, University of Electronic Science and Technology of China, Zhongshan, Guangdong 528400, China; ^2^Zhongshan Hospital of Traditional Chinese Medicine, Guangzhou University of Chinese Medicine, Zhongshan, Guangdong 528401, China

## Abstract

The Janus kinases (JAKs) consist of four similar tyrosine kinases and function as key hubs in the signaling pathways that are implicated in both innate and adaptive immunity. Among the four members, JAK3 is probably the more attractive target for treatment of inflammatory diseases because its inhibition demonstrates the greatest immunosuppression and most profound effect in the treatment of such disorders. Although many JAK3 inhibitors are already available, certain shortcomings have been identified, mostly acquired drug resistance or unwanted side effects. To discover and identify new promising lead candidates, in this study, the structure of JAK3 (3LXK) was obtained from the Protein Data Bank and used for simulation modeling and protein-ligand interaction analysis. The ~36,000 Chinese herbal compounds obtained from TCM Database@Taiwan were virtually screened by AutoDock Vina docking program and filtered with Lipinski's Rules and ADME/T virtual predictions. Because of high occurrence of fake hits during docking, we selected 12 phytochemicals which have demonstrated modulating JAKs expressions among the top 50 chemicals from docking results. To validate whether these compounds are able to directly mediate JAK3 kinase, we have investigated the inhibitory activity using enzymatic activity assays, western blot, and HEK 293 cell STAT5 transactivity assays. The molecular analysis included docking and molecular dynamics (MD) simulations in order to investigate structural conformations and to explore the key amino acids in the interaction between JAK3 kinase and its putative ligands. The results demonstrated that Cryptotanshinone, Icaritin, and Indirubin exhibited substantial inhibitory activity against JAK3 kinase* in vitro*. The results also provide binding models of the protein-ligand interaction, detailing the interacting amino acid residues at the active ATP-binding domains of JAK3 kinase. In conclusion, our work discovered 3 potential natural inhibitors of JAK3 kinase and could provide new possibilities and stimulate new insights for the treatment of JAK3-targeted diseases.

## 1. Introduction

The Janus kinases (JAKs) are a family of four similar tyrosine kinases (JAK1, JAK2, JAK3, and TYK2) that function as key hubs in the signaling pathways and receptors for cytokines implicated in both innate and adaptive immunity [1, 2]. In recent years, JAKs have been identified as attractive therapeutic targets for inflammatory diseases in the fields of both biotechnology and pharmaceuticals. Among the four JAK family members, JAK3 is probably the more attractive target because it exhibits the greatest immunosuppression and causes the most profound effect in the treatment of inflammatory diseases [1, 3]. However, the current approved JAK3 inhibitors showed undesirable side effects including anaemia and neutropenia [4, 5].

Chinese herbal compounds have been recognized as a tremendous contribution to drug development because they are generally well tolerated and have better biocompatibility and diversity in molecular structure and bioactive substructures [6].

Thus, Chinese herbal chemicals could be developed as promising lead candidates for the inhibitors against JAK3. The structure of JAK3 was obtained from the Protein Data Bank (PDB ID code: 3LXK) and used for simulation modeling, and protein-ligand interaction analysis. The ~36,000 Chinese herbal compounds obtained from TCM Database@Taiwan [7] were virtually screened by AutoDock Vina docking program [8] and filtered with Lipinski's Rules [9, 10] and ADME/T virtual predictions [11].

Although docking simulation has been commonly used in virtual screening for drugs development, the docking results sometimes have been questioned because of high occurrence of fake top potent hits [12, 13]. It has been reported that selecting candidates from validated traditional Chinese medicines could effectively improve the success rate of drug development [14]. Therefore, to discover and identify the most potential candidates of JAK3 inhibitor, we carefully evaluated and selected 12 Chinese herbal chemicals among the top 50 chemicals from docking results. The selected 12 phytochemicals, which modulate the expression of JAKs and have thus been effective in the treatment of inflammatory diseases, have been demonstrated using western blot analysis or immunohistochemistry assays through searches on the Web of Science Core Collection. These phytochemicals were tested for their ability to bind to JAK3 using biological activity assays. Subsequently, based on the results of the bioactivity assays, the 3 compounds with the highest JAK3 affinity (IC_50_<100*μ*M) were selected and their interaction with JAK3 kinase was investigated by docking computational again and evaluated by molecular dynamics (MD) simulations to provide further insight into the probable JAK3 protein-ligand interactions (the 12 chemicals were listed in Table 1).

## 2. Materials and Methods

### 2.1. Reagents and Materials

Twelve Chinese herbal compounds modulating the JAK kinase were selected from the first-round docking results and the published literature, purchased and tested, as detailed in Table 1.

Monoclonal Antiphosphotyrosine–Peroxidase antibody raised in mouse (catalog # A5964), synthetic polypeptide poly(Glu, Tyr), sodium salt, 20-50 kDa (catalog # P0275), globulin bovine serum albumin (BSA) (catalog #A3059), DL-dithiothreitol (catalog #D9779), adenosine-50-triphosphate disodium salt hydrate (ATP) (catalog # A2383), and sodium orthovanadate (Na3VO4) (catalog #S6508) were purchased from Sigma-Aldrich GmbH, Germany. JAK3 kinase domain (amino acids 781 to 1124) was purchased from Millipore, UK (catalog #D8EN006U-H). 2-[4-(2-hydroxyethyl)piperazin-1-yl] ethanesulfonic acid (HEPES) (catalog # L1613) was obtained from Biochrom GmbH, Germany. MgCl_2_·6H2O (catalog # 105833) was purchased from Merck KGA, Germany. 3,3',5,5'-tetramethylbenzidine (TMB) peroxidase reagent (catalog # 555214) was obtained from BD Biosciences Europe. Nunc MaxiSorp® microtiter plates (catalog # 442404) were obtained from Fisher Scientific GmbH, Germany. Plasmids pGL4.52-luc2P/STAT5RE (catalog # E465A) and pRL-TK (catalog # E2241) and the dual luciferase assay kit (catalog # E1980) were purchased from Promega, USA. Lipofectamine 2000 (catalog # 11668019) was obtained from Invitrogen, USA. HEK 293 cells were purchased from the Cell Bank of Type Culture Collection of Chinese Academy of Sciences. Ultrapure water produced using a Millipore Synergy UV water purification device was used throughout the study. All reagents were of ultrapure grade.

### 2.2. JAK3 Enzymatic Activity Assay

Kinase activity was determined using an ELISA-based assay according to the procedure reported by Bauer [27].

Kinase buffer (KB), containing 100 mM HEPES, 10 mM MgCl_2_, 4 mM dithiothreitol, 0.1 mM Na_3_VO_4,_ and 5* μM* ATP, was prepared in ultrapure water, the pH adjusted to 6.8–6.9 and used to prepare JAK3 kinase solution at a concentration of 100 ng/mL and for dilution of the test compounds. The synthetic polypeptide poly (Glu, Tyr) served as the substrate for kinase JAK3. It was dissolved in PBS at a concentration of 10 *μ*g/mL and then each well of a 96-well microplate was coated by adding 100 *μ*L of this working solution. The plate was sealed and incubated overnight at 4°C. Into the wells of the assay plate, 50 *μ*L of either pure KB, KB containing JAK3 kinase (100ng/mL of final concertation), or KB containing JAK3 kinase and a test compound were added followed by incubation at 37°C for one hour. One hundred *μ*L of antiphosphotyrosine–peroxidase conjugated antibody (diluted 1:10 000) were added to each well of the plate, which was incubated at 37°C for an additional hour. Finally, TMB substrate reagent was added to the wells and incubated at room temperature for 5 min. The reaction was blocked by the addition of 25 *μ*L of 1 M sulfuric acid. The optical density (OD) of the wells was measured immediately at 450 nm using a microplate reader. The quantity of kinase substrate that was phosphorylated was proportional to JAK3 kinase activity. CP-690,550 was selected as positive control. The OD of self-stimulation (STIM) without inhibitors indicated the maximum phosphorylation, and the OD of plain KB (NSB) was considered as negative control. The inhibition activity is calculated as the following equation: Inhibition(%)= 100%×[1-(OD_Sample_-OD_NSB_)/(OD_STIM_-OD_NSB_)].

### 2.3. Western Blot Analysis

To validate whether the hit compounds are able to mediate JAK3 kinase in cells, we investigated the JAK3 phosphorylation using western blot* in cellulo.*

HEK 293 cells were seeded in 6-well plates at a density of 5×10^4^ cells/well in 2 ml cell culture per well. Cells were treated with Cryptotanshinone, Icaritin, and Indirubin at 25 and 50 *μ*M concentrations for 1 h prior to stimulation with 20 ng/mL of IL-2 for 3 h and were harvested for phoso-JAK3 signaling determination. Cells without drug treatment were selected and used as control group. The above analyses were performed in three independent experiments.

Cells of all the groups were lysed and total proteins were extracted using RIPA lysis buffer (Beyotime, Shanghai, China) plus the PhosStop (Roche, Indianapolis, IN, USA) phosphatase inhibitors and Complete Ultra protease inhibitors (Roche). Equal amounts of protein were used to perform electrophoresis on a 12% SDS-polyacrylamide gel and subsequently transferred to polyvinylidene difluoride (PVDF) membranes (Millipore, Billerica, MA, USA). After blocking with 5% BSA in Tris-buffered saline (TBS) containing 0.1% Tween-20 for 1 h at room temperature, the membranes were incubated overnight at 4°C with the primary antibodies (1:1000). The primary antibodies, rabbit monoclonal phospho-JAK3, were purchased from Cell Signaling Technology (BSN, USA). Membranes were washed three times using Tris-buffered saline Tween-20 (TBST) for 5 min and then incubated for 2 h at room temperature with horseradish peroxidase-conjugated secondary antibody (1:1,000; Boster, Wuhan, China). After three TBST washes, the target proteins bands were detected by enhancing Pierce ECL Plus (ThermoFisher, Rockford, IL, USA) reagents and exposed to X-ray film (Eastman Kodak, Rochester, NY, USA) for the visualization. GAPDH was used as the internal loading control for protein normalization.

### 2.4. Stat5 Transactivation Activity

Activation of JAKs can induce constitutive expression of STAT5 that can be detected during an inflammatory response. STAT5 is one of the principal downstream signaling proteins induced by activated JAK3 and serves a critical role in inflammation and cell survival. To investigate STAT5 regulation by the Chinese herbal compounds, transcriptional activity of STAT5 was quantified by the use of a pSTAT5-Luc plasmid.

HEK 293 cells were transiently cotransfected with STAT5-responsive luciferase reporter pGL4.52-luc2P/STAT5RE/Hygro (firefly luciferase) and pRL-TK (renilla luciferase) plasmids, the latter acting as a transfection control [28, 29]. HEK 293-STAT5-Luc cells that were proliferating exponentially were seeded into the wells of a 24-well plate at a concentration of 5×10^4^ cells/well. Twenty-four h after transfection, the cells were preincubated with an appropriate concentration of each Chinese herbal compound for 1 h. The cells in each well were then stimulated with 20 ng/mL of IL-2 for an additional 3 h, after which passive lysis buffer was added (50 *μ*L/well). The plate was then incubated for 15 min on a shaking platform. A dual luciferase assay kit was used to measure luciferase expression using a GloMAX 20/20 luminometer. Transcriptional activity corresponded to the ratio of firefly luciferase luminescence to that of renilla luciferase.

### 2.5. Computational Molecular Simulation

The Chinese herbal compounds were employed to explore their interactions with JAK3 protein according to Rashid's methods [30].

#### 2.5.1. JAK3 Kinase Protein Structures

The crystal structure of JAK3 kinase (PDB ID: 3LXK) was obtained from the Protein Data Bank (PDB) (http://www.rcsb.org). Energy minimization of the structure was employed using Amber's force field and conjugate gradient algorithms provided in UCSF Chimera 1.5.6 software [31].

#### 2.5.2. Ligand Preparation

In the first-round docking simulation, the Chinese herbal compounds (~40,000) were obtained from the TCM Database@Taiwan provided by Dr. Chen's, and employed using AutoDock 4.2 tools for JAK3 structure-based virtual screening.

In the second-round docking simulation, three Chinese herbal compounds identified as inhibiting JAK3 demonstrating high JAK3 affinity (IC50<100 *μ*M) were selected and compared with CP690,550 (2-cyano-3-(3,4-dihydroxyphenyl)-N-(phenylmethyl)-2-propenamide), a positive control to investigate the ligand-binding interaction of JAK3–PTK domains.

#### 2.5.3. Docking Simulations

Molecular docking simulations of JAK3 kinase were conducted using AutoDock 4.2 and AutoDock Vina software, (Scripps Research Institute) according to published methods [32, 33]. It was anticipated that the docking results should provide the primary binding conformations of JAK3 with its ligands prior to MD simulation, after which optimized binding sites and interactions would be ascertained. Firstly, all hydrogen atoms had to be added to either the protein or the ligands. However, only polar hydrogen coordinates are utilized during docking simulations. Gasteiger-Marsili atomic charges are incorporated into the calculations of electrostatic interactions and desolvation energies by AutoDock. During docking simulations all rotatable bonds of the chemical ligands were allowed free choice of torsional degrees of freedom. Rigid receptor and flexible ligand were assumed in the docking simulations. A grid 60 Å × 60 Å × 60 Å was defined on the structure of JAK3 kinase in order to generate a grid map. Each docking simulation was repeated 100 times and employed empirical free energy and a Lamarckian genetic algorithm with the following parameters: the population was set at 150 randomly selected individuals, a maximum of 27,000 generations, a mutation rate of 0.02, crossover rate of 0.80, and energy evaluation of 2.5 × 10^6^. The docked complex conformation with lowest binding energy for each receptor was selected for further MD analysis. The hydrophobic and electrostatic interactions of docked complexes were mapped using Ligplot+ [34] and Discovery Studio 3.5 visualizer tools (http://accelrys.com/events/webinars/discovery-studio/index.php).

#### 2.5.4. MD Simulations

The MD simulations were performed using Groningen Machine for Chemicals Simulations (GROMACS) 4.6.5 software [35], using a GROMOS96 43A1 force field [36] and SPC/E molecular water model [37, 38]. The topology file and force field parameters of the chemical ligands were generated using PRODRG software [39]. The MD simulation was constructed in a cube with dimensions of 120 Å along each edge. Sodium or chloride ions were added to the MD simulation to neutralize the net charge. The SHAKE algorithm was utilized to fix the lengths of the bonds containing hydrogen atoms [40] and Particle Mesh Ewald (PME) used to calculate long-range electrostatic interactions. Before performing the MD simulations, energy minimization was performed with a maximum force per complex not greater than 1000 kJ/mol Å. Constant temperature and pressure equilibrations of complexes were performed over 1000 ps. Finally, the constructed MD simulations were performed over 20 ns at a temperature of 300 K and 1 atmosphere pressure. Coordinates were recorded every 100 ps.

After conducting the MD simulations, the conformations, trajectories, and behaviors of each simulation were analyzed using VMD [41], PyMol (http://www.pymol.org) [42] and GROMACS software.

#### 2.5.5. Statistical Analysis

Statistical analysis was performed using GraphPad Prism Version 5.01. Data are expressed as mean ± standard error of the mean (SEM). The significance of differences between groups was estimated by one-way ANOVA, adjusting for repeated measures with Dunnett's multiple comparison tests.

## 3. Results

### 3.1. JAK3 Enzymatic Activity Assay

**Figure 1:**
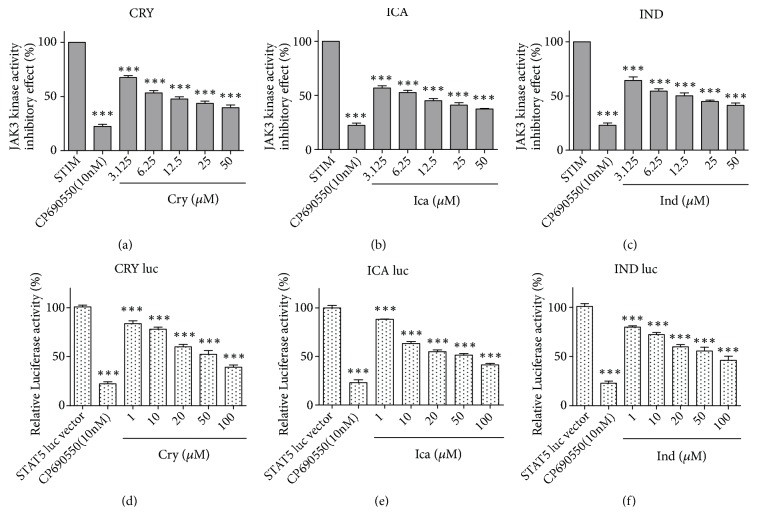
*Inhibition of JAK3 phosphorylation activity (a–c).* Inhibition of JAK3 (%) by 3 Chinese herbal compounds: (a) Cryptotanshinone (CRY), (b) Icaritin (ICA), and (c) Indirubin (IND), at various concentrations. JAK3 phosphorylation was measured using monoclonal antiphosphotyrosine–peroxidase conjugated antibody. Data represent means ± SEM of three independent experiments. The self-simulation (STIM) without inhibitors indicated the maximum phosphorylation. Compound CP690,550 was selected as positive control.* Inhibition of cellular JAK3-mediated STAT5 activity (d–f).* Inhibitory effect of treatment with 3 Chinese herbal compounds on luciferase activity of STAT5 at various concentrations. HEK 293 cells were transfected with a STAT5-dependent luciferase reporter then treated with herbal compounds for 1 h prior to conducting the luciferase reporter assay. Data represent means ± SEM of three independent experiments. *∗∗∗*p <0.001 versus control group; significance was determined using one-way ANOVA.

Based on the first-round docking results and the effects of Chinese herbal compounds on the JAKs/STATs pathway published in the literature, we selected and investigated the effect of 12 compounds on JAK3 phosphorylation activity. Among them, 3 compounds, Cryptotanshinone, Icaritin, and Indirubin, demonstrated significant reduction in JAK3 activity (IC_50_<100 *μ*M). The results of the 3 natural compounds showing highest affinity are shown in Figure 1 and S2.

### 3.2. JAK3 Enzymatic Activity Assay

**Figure 2:**
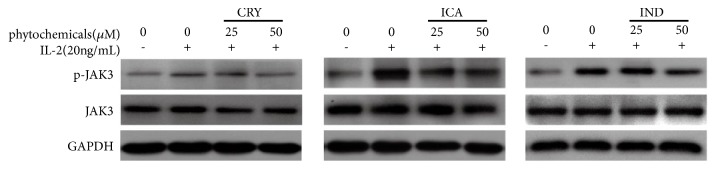
*Effects of hit Chinses herbal chemicals on JAK3 phosphorylation.* IL-2 induced phosphorylated JAK3 protein in HEK-293 cell line was determined by western blot after treatment with phytochemicals at the indicated concentrations for 2 h prior to stimulation with IL-2 for 1 h. GAPDH was used as the internal control for protein normalization.

The 3 hit compounds, Cryptotanshinone, Icaritin, and Indirubin, demonstrated directly inhibitory effects on the enzyme activity. Therefore, in this work we measured the changes of phoso-JAK3 content in HEK 293 cells by western blot. The result showed that 3 Chinses herbal compounds, Cryptotanshinone, Icaritin, and Indirubin, attenuated IL-2-induced JAK3 phosphorylation in HEK 293 cell line in Figure 2.

### 3.3. SATA5 Transactivation Activity

#### 3.3.1. Luciferase Experiments

Inhibition of JAK3 phosphorylation activity would be expected to lead to a decrease in downstream STAT5 phosphorylation levels. Thus, we sought to investigate the ability of the hit compounds to inhibit JAK3 signaling in human cells using a luciferase reporter assay. HEK 293 cells transfected with the luciferase reporter gene driven by a promoter containing multiple copies of the STAT5 response element were used in this study. The transcriptional activity of STAT5 was quantified by measuring the luciferase activity of cell lysates using a luminometer. We performed a dose response analysis of the 3 Chinese compounds in their ability to attenuate IL-2-induced STAT5 signaling (Figure 1). 

The compounds inhibited IL-2 induced luciferase activity in a dose-dependent manner. Although the other three compounds also inhibited IL-2-induced luciferase activity, the potency of their inhibition was approximately 1% that of CP- 690,550.

### 3.4. Docking Simulation

**Scheme 1:**
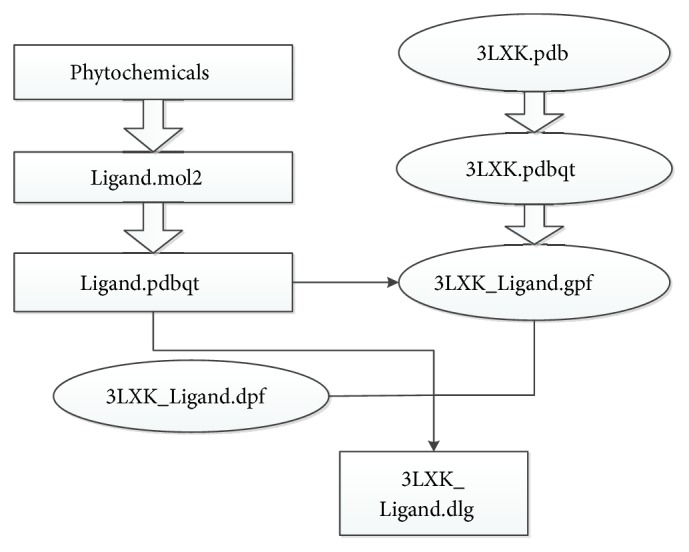
The workflow of the docking simulation.

The workflow of the docking simulation is outlined in Scheme 1. The molecular model of JAK3 for docking analysis was constructed using the reported X-ray cocrystal structure of JAK3 using the reference inhibitor CP-690,550. The binding site of JAK3 was defined to be within 3Å of the bound inhibitor, situated at the ATP-binding pocket of the JAK3 phosphorylated tyrosine kinase (PTK) domain.

On the first round of virtual screen, the phytochemicals of high docking scores were filtered using Lipinski's Rules of five and ADME/T virtual predictions. As phytochemicals are derived from natural plants, one or two violations of Lipinski's Rules were permitted [43]. The chemicals with toxicity under grade 2 of ADME/T virtual predictions were permitted [11]. The top 50 chemicals were listed in S1.

**Figure 3:**
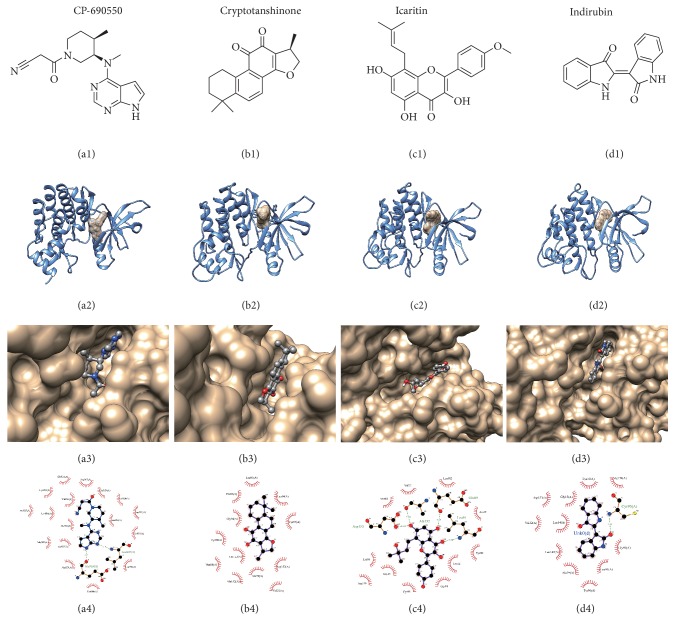
*Analysis of Chinese herbal compound binding to JAK3 kinase.* (a) CP-690550; (b) Cryptotanshinone; (c) Icaritin; (d) Indirubin. JAK3 kinase is depicted in marine-blue ribbon. Chemicals are shown as sticks & balls and transparent solids. JAK3 protein-ligand interactions were mapped using Ligplot+. CP-690,550 was the positive control.

Based on the results of the bioassays, the 3 compounds, Cryptotanshinone (CRY), Icaritin (ICA), and Indirubin (IND), with the highest bioactivity, were investigated by docking computation using AutoDock 4.2 again. Contact between compounds and the JAK3 binding pocket was analyzed using LigPlus 2.1 and Discovery Studio Viewer 3.5. Docking analysis results indicated that the interaction of the 3 herbal compounds could be situated in the binding-pocket region of JAK3. In the analysis, Icaritin formed hydrogen bonds with the same kinase domain (KD) residues as reference compound CP-690,550, including Glu903 and Leu 905, whereas Indirubin formed hydrogen bonds with KD residue Cys909. Cryptotanshinone could not form hydrogen bonds. Critical residues and interactions within the ATP-binding domain of JAK3-ligand complex are listed in Table 2. The lowest energy binding pose and the protein-ligand interactions are shown in Figure 3.

### 3.5. Molecular Dynamic Simulation

**Figure 4:**
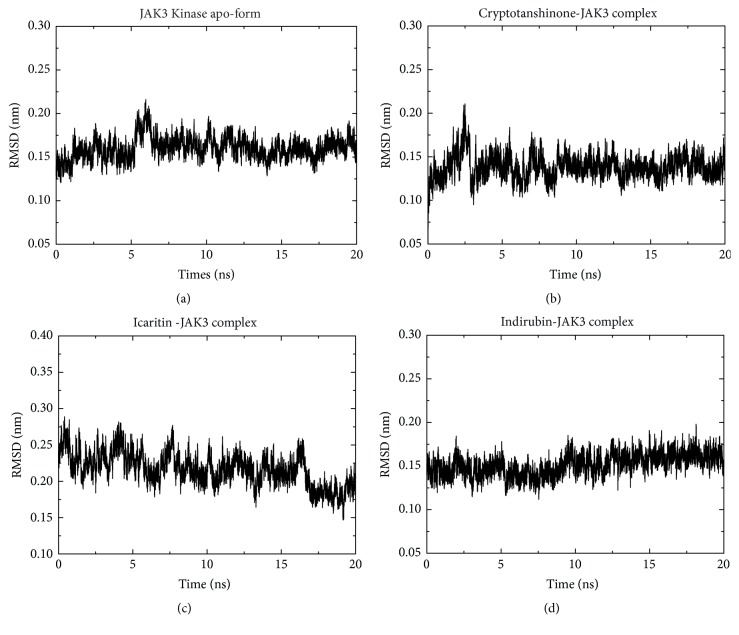
*Plots demonstrating JAK3 kinase-ligand stability.* RMSD versus time (20ns) graph through each MD trajectory file. (a) JAK3 Kinase apo-form; (b) Cryptotanshinone-JAK3 complex; (c) Icaritin -JAK3 complex; (d) Indirubin-JAK3 complex.

To investigate the stability of each JAK3-ligand complex, binding conformations of optimally docked complexes were studied through MD simulation assays. A least squares fit of the binding complexes was used in each case in the calculation of the root-mean-square deviations (RMSD). The RMSD for each complex was calculated over 5 ns. The output plots displayed RMSDs for each JAK3-ligand structure in its minimized, equilibrated state. MD simulation analysis indicated that the backbone RMSD profile for JAK3-compound complexes was quite stable (1.5–3 Å) throughout the MD trajectories, demonstrating stability of complexes during the MD simulations (Figure 4). The converging behavior indicated by these results provides credibility to the docking results.

## 4. Discussion

JAK kinases perform pivotal roles in inflammatory and oncological disorders [44]. Several JAK inhibitors have been developed for the treatment of inflammatory disease such as rheumatoid arthritis (RA) and autoimmune disorders such as inflammatory bowel disease and psoriasis [1, 3].

Unlike other JAK family members, the expression of JAK3 is mostly in hematopoietic cells where it exclusively associates with cytokine receptors bearing the common gamma-chain (*γ*c) subunit, enabling JAK3 to specifically operate in immune cells [45]. Consequently, JAK3 represents an attractive target for the treatment of inflammatory immunosuppression [46]. The JAK3 inhibitor Tofacitinib has been approved in US for treating RA patients [47].

Although many JAK3 inhibitors are already available, certain shortcomings have been identified, mostly acquired drug resistance or unwanted side effects [48, 49]. Physiological environments are more complicated than* in vitro* experimental conditions and unpredicted toxic effects can arise from the use of a potent inhibitor [50]. For example, European regulatory agencies did not approve Tofacitinib because of concerns over efficacy and safety [49]. Animal studies indicated the occurrence of carcinogenesis, mutagenesis, and impairment of fertility (XELJANZ prescribing information, Labeling.Pfizer.com). Thus, the development of novel JAK3 inhibitors or the application of medicines that inhibit JAK3 with limit side effects is required.

In this study, we performed an inhibition assay to determine the inhibitory activity of 12 phytochemicals from Chinese herbs against JAK3, using a cell-free enzyme assay and an HEK 293 cell pSTAT5 transactivity assay. 

The kinase and transactivity assays revealed that the majority of 12 test compounds did not exhibit kinase activity (IC_50_<100*μ*M) that qualified them as a “hit”. Some exhibited only limited activity while others showed no direct activity at all. Thus, these nonhit compounds probably modulate the JAK/STAT pathway through other mechanisms or factors. However, 3 compounds showed direct inhibition of JAK3 kinase* in vitro*. These results indicate that the IC_50_ of Cryptotanshinone was* ca.* 25 *μ*M, Icaritin* ca.* 20 *μ*M, and Indirubin* ca.* 50 *μ*M.

These compounds also demonstrated inhibition at low concentration in cell-based assays. There might be two reasons for this. Firstly, in cell culture conditions, there is a dynamic balance between kinase and phosphatase, whereas the kinase catalytic reaction persists* in vitro* until the substrates are saturated. The compounds in low concentration could disrupt this balance. Secondly, the compounds might affect STAT3 phosphorylation through other mechanisms. However, the nonhit compounds did not demonstrate activity either in the kinase assay or cell-based assays.

There is a tradition in Chinese medicine of use of the herbs containing the hit phytochemicals since ancient times, demonstrating a record of safety and efficacy [6, 51]. Thus, our study indicated that the hit compounds and their original Chinese herbs provide efficacy in the treatment of inflammatory diseases and immune disorders via direct interaction with JAK3 kinase.

To investigate the structural conformations and to explore the key amino acids involved in the interaction between JAK3 kinase and the hit ligands, we performed computational calculations using docking and molecular dynamics simulations.

In previous reports, structure-based computational molecular simulation has been used to identify candidate compounds able to inhibit JAK3 phosphorylation [52, 53]. The JAK3 ATP-binding pocket domain structure was used for computational chemical assays of small molecules. To explore models of compound binding to the JAK3 binding-pocket domain, computational modeling was conducted.

ATP-induced phosphorylation of Tyr on the JH1 domain of the JAK3 protein is critical for the function and modulation of JAK3 kinase [54]. The ATP-binding site of JAK3 kinase consists of a narrow and hydrophobic cleft located between the N- and C-lobes of the kinase domain (KD), the two lobes linked together by a binding-pocket region consisting of a hydrogen bond donor and acceptor residues from the protein backbone. The key factor in determining kinase inhibitory activity is the identity of residues in the glycine, hinge, and activation loops which control access of the inhibitor to the hydrophobic pocket. For this reason, we evaluated the magnitude of JAK3 inhibition of the 3 compounds targeting the ATP-binding pocket domain using computational assays.

Docking strategies generate binding or affinity scores for different sites and poses on the target protein. Although docking has been popularly applied in drug discovery, some docking results show high dock scores that are not well correlated with actual bioactivity, also failing in MD simulation [13]. To enhance the credibility of our docking results, we also performed MD simulations, the results of which demonstrated stable and converged behavior of complexes throughout the MD trajectories.

The docking simulation results of the 3 compounds using AutoDock 4.2, which are slightly lower than that using AutoDock Vina, showed a high dock score (the binding energy of Cryptotanshinone to JAK3 kinase was 9.2 kcal/mol, Icaritin 9.3 kal/mol, Indirubin 9.1 kcal/mol and CP-690,550 8.8 kcal/mol), indicating that the 3 phytochemicals are indeed excellent potential inhibitors. The poses found in this modeling approach indicated that the 3 Chinese herbal compounds could possibly bind to the JH1 domain of JAK3. The refined model predicted that these compounds bind at the specific site at which the binding-pocket residues are located within the JAK3 JH1 domain (Table 2). The model also predicted that Icaritin can form a number of hydrogen bonds with nearby amino acid residues, including Glu903, Leu905, Ala966, and Asp967, and that Indirubin can form a hydrogen bond with Cys909 (Figure 3). The results also indicate that the amino acid residues Leu828, Gly829, Lys830, Val836, Ala853, Met902, Glu903, Tyr904, Leu905, Gly908, Cys909, Leu956, Ala966, and Asp967 are those mostly involved in the interaction between JAK3 kinase and the compounds and are possibly the most important residues in the binding-pocket for protein-ligand interaction.

Thus, by combining bioactivity assays and computational research, the present study identified 3 Chinese herbal compounds that could serve as potential candidates for JAK3 kinase inhibition and JAK3-targeted diseases.

## 5. Conclusions

In conclusion, we have discovered 3 potential natural inhibitors of JAK3, Cryptotanshinone, Icaritin, and Indirubin. Our work could provide new possibilities and stimulate new insights for the treatment of JAK3-targeted diseases.

## Figures and Tables

**Table 1:** Chinese herbal compounds analyzed.

No.	Phytochemical	Plant Species	Reference
1	Andrographolide	*Andrographis paniculata* L.	[15]
2	Arctigenin	*Fructus Arctii *L.	[16]
3	Baicalein	*Scutellaria baicalensis *Fisch.	[17]
4	Berbamine	*Berberis amurensis* Rupr.	[18]
5	Cryptotanshinone	*Salvia miltiorrhiza *Bunge.	[19]
6	Curcumin	*Curcuma longa *L.	[20]
7	Ginkgolide B	*Ginkgo biloba* L.	[21]
8	Icaritin	*Epimedium grandiflorum *C. Morren	[22]
9	Indirubin	*Strobilanthes formosanus *Moore	[23]
10	Quercetin	*-*	[24]
11	Salvianolic acid B	*Salvia miltiorrhiza *Bunge.	[25]
12	Ursolic acid	*-*	[26]

**Table 2:** Details of interactions between compounds and the JAK3 ATP-binding site.

JAK3 binding region residues	Nature of interaction			
	CP690550	Cry	Ica	Ind
Leu828	Van der Waals	VDW	Electrostatic	Electrostatic
Gly829	Electrostatic	-	VDW	VDW
Lys830	Electrostatic	-	VDW	VDW
Gly831	Electrostatic	-	-	-
Gly834	Electrostatic	-	-	-
Ser835	Electrostatic	-	-	-
Val836	Electrostatic	VDW	VDW	VDW
Ala853	VDW	VDW	VDW	VDW
Lys855	VDW	-	-	-
Val884	VDW	-	VDW	-
Met902	VDW	VDW	VDW	-
Glu903	H-Bond	Electrostatic	H-Bond	-
Tyr904	VDW	VDW	VDW	VDW
Leu905	H-Bond	Electrostatic	H-Bond	VDW
Pro906	-	VDW	-	-
Gly908	-	VDW	Electrostatic	Electrostatic
Cys909	VDW	VDW	VDW	H-Bond
Arg953	VDW	-	Electrostatic	VDW
Asn954	VDW	-	Electrostatic	-
Ile955	VDW	-	-	-
Leu956	VDW	VDW	VDW	VDW
Ala966	VDW	VDW	H-Bond	-
Asp967	VDW	VDW	H-Bond	Electrostatic

## Data Availability

The authors declare that the data supporting the findings of this study are available within the article and the supplementary information file.

## Supplementary Materials

Supplementary Materials Table S1: top 50 docking results.
