# Supplementary material for: Identification of Chinese Herbal Compounds with Potential as JAK3 Inhibitors
Source: Evid Based Complement Alternat Med. 2019 Apr 9;2019:4982062. doi: 10.1155/2019/4982062 (PMC6481137; doi:10.1155/2019/4982062)
Supplement: Supplementary Materials — Table S1: top 50 docking results. [file 4982062.f1.pdf]

S1. Top 50 Docking Results

Rank	Ligand Name	Docking Scores	xLogP	H-bond donors	H-bond acceptors	Molecular weight	Rotatable bonds	Rat Acute Toxicity LD50 mol/kg	Acute Oral Toxicity
1	Schizotenuin F	11.8	3.48	5	12	551.48	12	2.24	3
2	Glucopyranoside	11.6	-0.03	4	10	321.33	7	2.8831	4
3	Emodin-8-beta-D-glucoside	11.5	0.96	6	10	432.381	3	2.5573	4
4	Icaritin	11.1	4.96	3	6	368.385	4	2.9476	3
5	Isochlorogenic acid A	11	1.42	6	12	515.447	9	2.4467	4
6	Nymphaeol A	10.9	5.52	4	6	424.493	6	3.1399	4
7	Lonijaposides A	10.8	-3.91	4	14	578.547	13	2.4583	4
8	Salvianolic acid A	10.8	3.01	6	10	493.444	9	2.3482	4
9	Trifolirhizin-6''-O-malonat	10.7	0.51	3	13	531.446	7	2.5318	4
10	(+)-Gallocatechin	10.6	1.08	6	7	306.27	1	1.87	3
11	Cryptotanshinone	10.5	3.83	0	3	296.366	0	2.188	3
12	Verbascoside	10.5	-0.45	9	15	624.592	11	1.939	3
13	Berbamine	10.5	6.24	1	8	608.735	3	2.671	3
14	3-Galloylcatechin	10.4	2.54	7	10	442.376	4	2.6643	4
15	Salvianolic acid B	10.4	1.62	7	16	716.604	14	3.2895	3
16	Echinacoside	10.3	-2.15	12	20	786.733	14	1.9237	3
17	Indirubin	10.2	2.18	1	4	262.268	1	2.2017	3
18	Orientin	10.2	0.003	8	11	448.38	3	2.3664	4
19	Isocrenatoside	10.1	0.02	8	15	622.576	8	2.2693	3
20	Gallotannin	10.1	_	_	_	2073.541	_	2.5311	3
21	Delphinidin-3-O-glucoside	10.1	-0.1	8	12	464.379	4	2.4148	3
22	Pulmatin	10	1.3	5	9	416.382	3	2.5573	3
23	Eriocitrin	10	-1.4	9	15	596.538	6	2.4984	3

24	Ginkgolide B	10	-2.38	3	10	424.402	1	2.497	3
25	Isorhoifolin	10	-0.02	8	14	578.523	6	2.4367	3
26	Forsythoside A	10	-0.23	9	15	624.592	11	2.2295	3
27	Hellicoside	10	-1.16	11	17	656.59	12	2.1005	3
28	2'-Methoxykurarinone	9.9	5.9	2	6	452.547	8	3.1774	3
29	Mangiferin	9.9	-0.16	8	11	422.342	2	2.3664	4
30	Luteolin 7-galactoside	9.9	0.19	7	11	448.38	4	2.3869	3
31	Isosalvianolic acid C	9.9	3.38	6	10	492.436	7	3.4184	2
32	Melittoside	9.9	-4.64	10	15	524.472	7	2.6373	4
33	3,5-Dicaffeoylquinic acid	9.9	1.42	6	12	515.447	9	2.4214	3
34	Baicalein	9.9	2.68	3	5	270.24	1	3.02	2
35	Isoacteoside	9.8	-0.23	9	15	624.592	11	2.0947	3
36	Arctigenin	9.8	2.22	1	6	372.417	7	2.5844	3
37	Nystose	9.8	-5.08	14	21	666.579	13	1.3683	3
38	Narirutin 4'-glucoside	9.8	-2.9	11	19	742.68	9	2.0375	3
39	Lucidumoside A	9.8	0.54	5	12	526.535	12	2.585	3
40	Quercetin	9.8	1.68	5	7	302.238	1	3.02	2
41	Lobetyolinin	9.7	-1.64	9	13	558.577	12	1.6964	2
42	Curcumin	9.6	2.3	2	6	368.385	8	2.5468	3
43	Isoangustone A	9.4	6.04	4	6	422.477	5	3.0645	3
44	Lycopic acid B	9.2	2.63	8	16	718.62	14	3.3595	2
45	Andrographolide	9	1.05	3	5	350.455	3	3.0943	3
46	Cynaroside	8.9	0.19	7	11	448.38	4	2.3869	3
47	Astringin	8.8	0.71	7	9	406.387	5	2.1382	3
48	Oleracein A	8.8	-2.01	7	11	503.46	6	2.3343	3
49	Ursolic acid	8.7	6.79	1	3	455.703	1	2.1645	3
50	Sanggenol A	8.7	5.92	4	6	424.493	6	3.3264	3

S2. The JAK3 Kinase Activity Inhibitory Effects of 12 Phytochemicals

No.	Phytochemical	Docking Results	JAK3 Kinase Activity Inhibitory Effect (%)					
			3.125μM	6.25μM	12.5μM	25μM	50μM	100μM
**1**	Andrographolide	*9*	75.82±2.86	68.84±2.16	68.35±1.06	59.44±1.78	60.33±2.08	60.72±3.86
**2**	Arctigenin	*9.8*	90.35±1.76	77.28±1.94	79.24±2.75	78.12±3.05	76.54±1.64	72.89±1.67
**3**	Baicalein	*9.9*	85.32±2.22	86.35±3.16	84.47±2.86	83.88±1.88	78.25±2.32	71.45±3.18
**4**	Berbamine	*10.5*	79.36±3.42	78.33±3.81	75.42±2.85	71.65±3.26	70.73±2.89	66.82±2.18
**5**	Cryptotanshinone	*10.5*	67.48±1.79	53.18±2.19	47.71±1.96	43.60±2.19	39.63±2.46	32.18±3.16
**6**	Curcumin	*9.6*	88.04±3.56	78.51±3.62	80.36±2.91	72.64±2.43	62.66±1.07	61.83±3.92
**7**	Ginkgolide B	*10*	100.31±2.61	98.62±4.06	96.34±2.89	91.85±3.71	91.67±2.99	88.19±2.84
**8**	Icaritin	*11.1*	56.84±2.10	52.73±2.07	45.17±2.04	41.09±2.23	37.43±0.55	34.81±2.62
**9**	Indirubin	*10.2*	64.39±3.24	54.45±2.18	50.14±2.74	44.85±1.40	42.28±2.25	38.47±3.05
**10**	Quercetin	*9.8*	86.45±2.37	81.76±2.98	82.67±3.28	75.06±3.33	72.82±2.75	71.67±2.66
**11**	Salvianolic acid B	*10.4*	82.85±3.83	83.20±1.85	81.64±2.48	82.60±3.76	78.54±4.51	80.28±3.45
**12**	Ursolic acid	*8.7*	101.36±3.78	102.81±4.24	98.37±1.96	85.25±1.26	87.36±4.26	75.06±2.98
